# Mapping neural activity patterns to contextualized fearful facial expressions onto callous-unemotional (CU) traits: intersubject representational similarity analysis reveals less variation among high-CU adolescents

**DOI:** 10.1017/pen.2020.13

**Published:** 2020-11-10

**Authors:** Shawn A. Rhoads, Elise M. Cardinale, Katherine O’Connell, Amy L. Palmer, John W. VanMeter, Abigail A. Marsh

**Affiliations:** 1 Department of Psychology, Georgetown University, Washington DC 20057, USA; 2 Interdisciplinary Program in Neuroscience, Georgetown University, Washington DC 20057, USA; 3 Independent Scholar; 4 Department of Neurology, Georgetown University Medical Center, Washington DC 20057, USA

**Keywords:** callous-unemotional traits, fearful faces, representational similarity analysis

## Abstract

Callous-unemotional (CU) traits are early-emerging personality features characterized by deficits in empathy, concern for others, and remorse following social transgressions. One of the interpersonal deficits most consistently associated with CU traits is impaired behavioral and neurophysiological responsiveness to fearful facial expressions. However, the facial expression paradigms traditionally employed in neuroimaging are often ambiguous with respect to the nature of threat (i.e., is the perceiver the threat, or is something else in the environment?). In the present study, 30 adolescents with varying CU traits viewed fearful facial expressions cued to three different contexts (“afraid for you,” “afraid of you,” “afraid for self”) while undergoing functional magnetic resonance imaging (fMRI). Univariate analyses found that mean right amygdala activity during the “afraid for self” context was negatively associated with CU traits. With the goal of disentangling idiosyncratic stimulus-driven neural responses, we employed intersubject representational similarity analysis to link intersubject similarities in multivoxel neural response patterns to contextualized fearful expressions with differential intersubject models of CU traits. Among low-CU adolescents, neural response patterns while viewing fearful faces were most consistently similar early in the visual processing stream and among regions implicated in affective responding, but were more idiosyncratic as emotional face information moved up the cortical processing hierarchy. By contrast, high-CU adolescents’ neural response patterns consistently aligned along the entire cortical hierarchy (but diverged among low-CU youths). Observed patterns varied across contexts, suggesting that interpretations of fearful expressions depend to an extent on neural response patterns and are further shaped by levels of CU traits.

Callous-unemotional (CU) traits are early-emerging personality features characterized by deficits in empathy, concern for others, and remorse following social transgressions (Frick & White, 2008). These traits, which encompass the core affective and interpersonal traits of psychopathy, are associated with severe and persistent disruptive behavioral problems in childhood and adolescence, including aggression and delinquency, and pose a high risk for persistent aggressive and criminal behavior in adulthood (Barry et al., 2000; Burke, Loeber, & Lahey, 2007; Pardini, 2006; Pardini & Frick, 2013; Salekin, Brannen, Zalot, Leistico, & Neumann, 2006; Vasey, Kotov, Frick, & Loney, 2005). Among the interpersonal deficits most consistently associated with CU traits are reduced behavioral and neurophysiological responsiveness to signs of others’ distress (e.g., fearful facial expressions) (Fanti et al., 2017; Jusyte, Mayer, Künzel, Hautzinger, & Schönenberg, 2015; Lozier et al., 2014; Marsh et al., 2008; Sebastian et al., 2014) as well as difficulty accurately interpreting these expressions (Dawel, O’Kearney, McKone, & Palermo, 2012; Marsh & Blair, 2008; Wilson, Juodis, & Porter, 2011). However, because most neuroimaging studies of facial expression responding in CU youths feature passive viewing paradigms, there is little direct evidence linking how these expressions are interpreted by high-CU youths to their anomalous neural responses. The present study is the first to investigate neural responses to fearful facial expressions in different interpretative contexts in high-CU adolescents. We employed a novel data-driven approach to explore how variations in neural responding across interpretive contexts correspond to variations in CU traits.

High levels of CU traits are observed in approximately one-third of children with clinically significant conduct problems (Frick & Viding, 2009; Mills-Koonce et al., 2015) and are consistently linked to atypical patterns of neural responding to a variety of fear-associated stimuli (Carré, Hyde, Neumann, Viding, & Hariri, 2013; Jones, Laurens, Herba, Barker, & Viding, 2009; Marsh et al., 2008). Such stimuli include fearful facial expressions (Marsh, 2016), which serve important social functions that include signaling internal states of distress to others (Horstmann, 2003), signaling vulnerability and appeasement to inhibit aggression or elicit care (Hammer & Marsh, 2015; Marsh, Ambady, & Kleck, 2005), and promoting social learning about environmental threats (Hooker, Germine, Knight, & D’Esposito, 2006; Olsson & Phelps, 2007). High-CU youths and adults exhibit atypical responses to fear or other negative emotions expressed by the face, voice, or body, including reduced autonomic responding, reduced fear-potentiated startle, and impaired aversive conditioning (Blair, 1999; Blair, Jones, Clark, & Smith, 1997; Fanti et al., 2017; Fanti, Panayiotou, Kyranides, & Avraamides, 2016; Kimonis, Fanti, Goulter, & Hall, 2017; Marsh et al., 2011; Muñoz, 2009; Rothemund et al., 2012). High-CU individuals also consistently show atypical patterns of neural activation while viewing fearful expressions, including reduced amygdala activation and striatum (Carré et al., 2013; Jones et al., 2009; Lozier et al., 2014; Marsh et al., 2008). However, less is known about where the stream of neural information processing of fearful faces in high-CU youths diverges from that of low-CU youths.

Information conveyed by faces is processed in distributed but overlapping systems of cortical and subcortical brain regions (Freiwald, Duchaine, & Yovel, 2016; Haxby et al., 2001; Ishai, 2008; Pessoa & Adolphs, 2010). This information is systematically transformed from early representations in visual cortex (e.g., distinct facial features) to more complex, identity-specific representations in fusiform gyrus (e.g., combinations of features, then further reduced to aggregated, feature-invariant information) hierarchically along a posterior–anterior axis. Human neuroimaging finds evidence of activation to emotional faces along this hierarchy, including the visual cortex, fusiform gyrus (FFG), amygdala, superior temporal cortex, and more frontal interpretative regions that include the medial prefrontal cortex (mPFC), and lateral prefrontal cortex (lPFC; including the inferior, middle, and superior frontal gyri) (Sabatinelli et al., 2011). The amygdala plays a key role in coordinating adaptive responses particularly to fearful expressions and other fear-linked stimuli through its reciprocal connections with other subcortical (e.g., hypothalamus, periaqueductal gray, midbrain) and cortical regions (e.g., insula, subgenual cingulate, and medial and lateral prefrontal cortex) (Blair, 2008; Davis & Whalen, 2001; Garvert, Friston, Dolan, & Garrido, 2014; Marsh, 2016; Robinson, Laird, Glahn, Lovallo, & Fox, 2010; Rosen & Donley, 2006). Furthermore, functional coupling among face-selective regions shows evidence for hierarchical clustering roughly into three subnetworks likely corresponding to processing individual identity (e.g., inferior occipital gyri, FFG), retrieval of semantic knowledge (e.g., lPFC, inferior parietal sulci, supramarginal gyri), and representation of emotional information (e.g., mPFC, orbital frontal cortex, insula, superior temporal sulci, temporal pole) (Zhen, Fang, & Liu, 2013). These regions receive input from and send modulatory feedback to lower-level sensory areas, which may enable the derivation of socioaffective meaning from emotional face stimuli.

But despite this relatively comprehensive understanding of the brain networks underlying emotional face processing, little is known about how patterns of responses to fearful expression vary as function of CU traits along the cortical hierarchy. This is in part because interpretations of fearful expression are unconstrained in most neuroimaging studies. Because of the variable functions that fearful expressions serve, when these expressions are viewed in decontextualized settings, the signal of these expressions could be interpreted in multiple ways – for example, as a readout of an internal state (i.e., the expresser is afraid for themselves), as an effort to inhibit aggression (i.e., signaling that the expresser is afraid of the perceiver), or as a cooperative social cue (i.e., the expresser is signaling that they are afraid for the perceiver). This fact has several implications. For one, different subsets of participants (e.g., those with high- versus low-CU traits) may tend to interpret these expressions differently, which could contribute to inconsistencies in patterns of neural, physiological, and behavioral responding observed in response to them. In addition, CU traits may be more closely associated with atypical responses to fearful expressions in specific interpretative contexts. For example, CU traits may be more closely associated with atypical responses to fear when it is interpreted as an aggression-inhibition cue, given links between CU traits and failures to inhibit aggression despite the distress is caused (Cardinale & Marsh, 2015). This suggests the importance of understanding variation in neurophysiological responses to fearful expressions as a function of how these expressions are interpreted in high-CU youths.

The present study investigated whether adolescents with high-CU traits differentially respond to fearful facial expressions in each of three distinct contexts. Our primary aim was to explore whether variations in patterns of neural activation associated with viewing fearful facial expressions in these different contexts map onto variation in CU traits. To pursue this question, we employed a paradigm that presented participants with contextually-ambiguous fearful expressions as well as expressions for which the interpretive context is constrained (i.e., participants are instructed to interpret expressions as readouts of internal states) and measured blood-oxygenated level-dependent (BOLD) signal in a sample of adolescents with varying levels of CU during fMRI.

To analyze the resulting data, we conducted both univariate analyses and multivoxel pattern analyses that examined how idiosyncratic neural response patterns mapped onto differential intersubject models of CU traits. This allowed us to both assess the consistency of our results with past results obtained from univariate analyses and test novel questions such as whether high-CU participants show neural response patterns that are more similar to one another than low-CU participants. We employed a novel technique called Intersubject Representational Similarity Analysis (IS-RSA; Chen, Jolly, Cheong, & Chang, 2020; Finn et al., 2020; Nguyen, Vanderwal, & Hasson, 2019; van Baar, Chang, & Sanfey, 2019) that assesses how interindividual variability in multivoxel brain activity patterns relates to individual differences in behaviors or traits using second-order statistics (Kriegeskorte, Mur, & Bandettini, 2008). This approach leverages between-participant differences in CU traits and enables us to treat participant-level phenotypic differences as signal instead of noise (Foulkes & Blakemore, 2018; van Baar et al., 2019). Specifically, we tested where multivoxel neural activity patterns associated with viewing fearful facial expressions in each of three contexts would be: 1) more similar among high scoring CU adolescents, but more dissimilar among all others; 2) more similar among low scoring CU adolescents, but more dissimilar among all others; and 3) more similar among adolescents who are very similar in their CU scores regardless of their absolute position on the scale, but more dissimilar for participants with dissimilar scores.

## 1. Method

### 1.1 Participants

The MRI sample consisted of 37 adolescents who varied in conduct problem severity and CU traits. Of the sample, 20 were assessed as exhibiting clinically significant CU traits; the remaining 17 adolescents did not exhibit significant levels of either CU traits or disruptive behavior. The sample included males and females aged 10 to 17 years old who were recruited from Washington, D. C. and surrounding areas through referrals, advertisements, fliers seeking both healthy children and children with conduct problems. All youths and a parent or guardian completed an initial screening visit to determine eligibility for the scanning portion of the study. During this visit, demographic and clinical assessments were conducted, including a test of cognitive intelligence (K-BIT, Kaufman & Kaufman, 2004), and assessments of clinical symptomology using the Strengths and Difficulties Questionnaire (Goodman & Scott, 1999), Child Behavior Checklist (CBCL; Achenbach, 1991), and the Inventory of Callous-Unemotional Traits (ICU; Frick & Ray, 2015; Kimonis et al., 2008) completed separately by parent and child. Participants were excluded based on the following criteria: full-scale IQ scores <80, history of head trauma, neurological disorder, parent report of pervasive developmental disorder, or magnetic resonance imaging (MRI) contraindications. In addition, no siblings were permitted to participate. Additional exclusion criteria for healthy controls included any history of mood, anxiety, or disruptive behavior disorders. Written informed assent and consent were obtained from children and parents before testing. Approval for all procedures was obtained from the Georgetown University Institutional Review Board.

Of this sample, four participants were excluded from analyses due to excessive motion during the MRI scanning and three participants were excluded from analyses for failing the attention checks during the task (>50% errors). The resulting sample consisted of 30 adolescents (*M*_*age*_ = 13.33, *SD* = 2.29; 14 females; see Tables 1a and 1b). All participants were native English speakers.

**Table 1a.** Demographic and behavioral characteristics

Characteristics	(*N* = 30)
Male:Female ratio	16:14
Age, mean (*SD*)	13.33 years (2.29)
IQ, mean (*SD*)	106.83 (13.92)
Race (n)	
White	15
Black or African American	9
Hispanic or Latino/a	3
Other	3
ICU Total, mean (*SD*)	35.82 (13.63)
Callous, mean (*SD*)	12.41 (6.44)
Unemotional, mean (*SD*)	8.73 (2.49)
Uncaring, mean (*SD*)	14.68 (5.96)
CBCL Externalizing, mean (*SD*)	18.93 (16.52)
Percent Accuracy (out of 24) during fMRI task, mean (*SD*)	85.83% (2.72)

**Table 1b.** Spearman *ρ* correlations among demographic and CU variables (*N* = 30)

	1	2	3	4	5	6	7
1. Age	–						
2. ICU Total	.10	–					
3. Callous	.03	.95**	–				
4. Uncaring	.16	.94**	.86**	–			
5. Unemotional	−.01	.65**	.54**	.54*	–		
6. CBCL^^^ (Externalizing)	−.02	.83**	.81**	.80**	.62**	–	
7. IQ (KBIT)	−.12	−.38*	−.39*	−.27	−.36	−.30	–

**p < 0.001, *p < 0.05; ^^^imputed one question item as average of remaining responses for one subject due to blank data

### 1.2 Inventory on callous-unemotional traits

In accordance with standard procedures, ICU scores were calculated using the highest item response from either the child or parent for each item, which reduces susceptibility to social desirability biases and optimizes accuracy across multiple contexts (Frick et al., 2003). Two subjects did not report an answer for a question item but their parents did, and one parent did not provide an answer for a question item but the subject did. For these instances, we used the only available item response. We also assigned “somewhat true” for two question items where one subject’s parent selected both “not at all true” and “somewhat true.” We then calculated a summary score of all item responses (ICU total) and each subscale (callous, uncaring, and unemotional) for each participant (Kimonis et al., 2008). Internal consistency was high for the ICU total scale (*α* = .92), callousness (*α* = .82), and uncaring (*α* = .90) subscales, but low-to-moderate for the unemotional subscale (*α* = .49), which is commonly observed among studies (Cardinale & Marsh, 2017).

### 1.3 fMRI task

**Figure 1.**
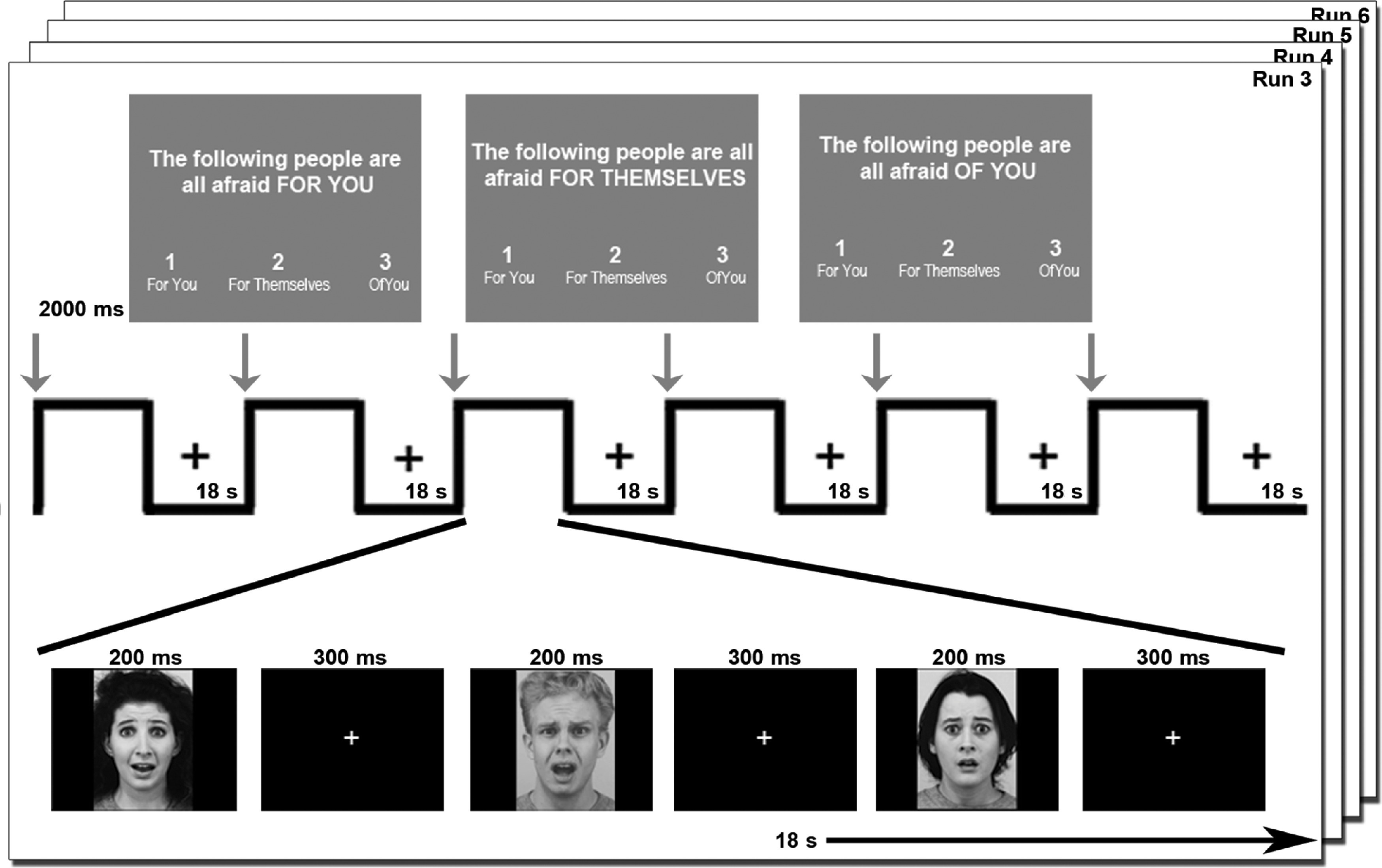
**Visualization of the fMRI Task.** During the first two runs of fMRI, participants viewed 18-s blocks of noncontextualized fearful facial expressions (200 ms) and fixation (300 ms) interleaved with 18-s blocks of fixation (not depicted). During the final four runs, participants again viewed 18-s blocks of fearful facial expressions (200 ms) and fixation (300 ms) followed by 18-s blocks of fixation. Prior to each face block, a sentence appeared for 2000 s indicating that the “following people are all afraid… ‘FOR YOU’, ‘FOR THEMSELVES’, or ‘OF YOU’.” Participants were asked to press one of three buttons that corresponded to the instruction as an attentional check.

We adapted an experimental task from Palmer and colleagues (2013), which comprised six runs. In the first two runs, participants viewed four 18-s blocks of rapidly presented fearful faces interleaved with four 18-s blocks of fixation for 3 min. During the fearful face blocks, faces were presented for 200 ms followed by a 300-ms fixation cross. The purpose of these baseline runs was to orient the participants to the fearful faces before presenting them in three experimental contexts. During the next four runs, participants again viewed 18-s blocks of rapidly presented fearful faces interleaved with 18-s blocks of fixation. During these runs, a 2000-ms cue appeared before each block that placed the faces in one of three contexts: “afraid of you,” “afraid for you,” or “afraid for themselves.” During this prompt, participants pressed one of three buttons corresponding to the condition to indicate that they had read and understood the context for the upcoming block. The interval between the offset of the cue and the onset of a face block pseudorandomly varied between 2500 and 3600 ms. Each contextualized block was presented twice per run for a total of six blocks of contextualized fearful faces interleaved with six blocks of fixation in each run. These final four runs of the task were 4 min 13 s each. The blocked design was selected to reduce cognitive load resulting from changing contexts (Figure 1).

Face stimuli included fearful facial expressions from 24 different exemplar individuals from the Karolinska face database (Lundqvist, Flykt, & Ohman, 1998), selected because the actors in this database were between the ages of 20 and 30, which is younger than most facial expression sets. Faces presented in grayscale were normalized for size and luminance. Each individual face was presented in only one of the three context condition blocks (i.e., six identities per context). The other six identities were presented within the first two (baseline) runs.

### 1.4 fMRI data acquisition

MRI data were acquired on a 3.0 T Siemens TIM Trio MRI scanner (Erlangen, Germany), located at the Georgetown University Center for Functional and Molecular Imaging. The first two functional scans consisted of 72 contiguous T2*-weighted echo planar imaging (EPI) whole-brain functional volumes while the final four functional scans consisted of 102 T2*-weighted EPI volumes. All functional scan contained the following parameters: repetition time (TR) = 2500 ms; echo time (TE) = 35 ms; flip angle =90°, 43 slices, matrix = 64 × 64; field of view (FOV) = 240 × 240 × 129 mm^3^; acquisition voxel size = 3.75 × 3.75 × 3.00 mm^3^. A T1-weighted high-resolution anatomical image was acquired for coregistration and normalization of functional images with the following parameters: TR = 1900 ms; TE = 2.52 ms; flip angle = 9°; 176 slices; FOV = 176 × 250 × 250 mm^3^; acquisition voxel size = 1.00 × 0.98 × 0.98 mm^3^.

### 1.5 fMRI preprocessing and first-level analysis

Preprocessing was performed using fMRIPrep 1.3.2 (Esteban et al., 2017, 2018), which is based on Nipype 1.1.9 (Gorgolewski et al., 2019; Gorgolewski et al., 2011) and included anatomical T1-weighted brain extraction, head-motion estimation and correction, slice-timing correction, intrasubject registration, and spatial normalization to the Montreal Neurological Institute 152 Nonlinear Asymmetric 2009c Template. The data were smoothed using a 6 mm^3^ full-width half-maximum Gaussian kernel prior to first-level beta parameter estimation. Using Statistical Parametric Mapping (SPM12; https://www.fil.ion.ucl.ac.uk/spm/), a general lineal model (GLM) was constructed for each participant across each of the six runs using boxcar regressors for each of the three instruction prompts, three fearful faces conditions with contexts, six motion parameters, one fearful faces condition without context, and a constant regressor for each run. The resulting GLM was convolved using a canonical hemodynamic response function and corrected for temporal autocorrelations using a first-order autoregressive model. Lastly, a standard high-pass filter (cutoff at 128 s) was used to exclude low-frequency drifts. Subject-level beta maps are available in Supplementary Data.

### 1.6 fMRI univariate analysis

We first conducted a series of whole-brain univariate multiple regression analyses in SPM12 to examine how neural activation to fearful facial expressions varied as a function of CU traits. For each model, we controlled for age, gender, and IQ. A family-wise error (FWE) corrected threshold of *p* < 0.05 with >10 voxels per cluster at the whole-brain level was applied to all analyses. To address *a priori* hypotheses regarding the role of the amygdala in fearful face processing, we also applied small volume correction using an anatomically-defined bilateral amygdala parcel from the Harvard Oxford Subcortical Atlas (Desikan et al., 2006; Goldstein et al., 2007; Makris et al., 2006) and conducted region-of-interest (ROI) analyses that averaged across voxels in the left and right amygdala.

Our first regression model examined the relationship between CU traits and activation during the first two baseline runs. The second regression analysis modeled how neural activation during the final four runs to fearful facial expressions varied across contexts (“afraid of you,” “afraid of you,” and “afraid for self”) and as a function of CU traits. We conducted each test for ICU total scores and subscale scores.

### 1.7 Searchlight intersubject representational similarity analysis (IS-RSA)

To test where multivoxel neural activity patterns associated with viewing contextualized fearful facial expressions corresponded to each of three models (high-CU alike, low-CU alike, and nearest neighbors), we employed IS-RSA (Chen et al., 2020; Finn et al., 2020; Nguyen et al., 2019; van Baar et al., 2019) using the NLTools package version 0.3.11 (Chang et al., 2019) in Python 3.5.2 and the CoSMoMVPA Toolbox version 1.1.0 in MATLAB 2017a. We first constructed three intersubject models (30 subjects × 30 subjects symmetrical matrices) based on ICU total scores, with each cell corresponding to the models’ prediction of a subject pairs’ neural pattern dissimilarity based on their CU scores. The first two models were generated using the Anna Karenina principle that “all low (or high) CU scorers are alike; each high (or low) CU scorer is different in his or her own way” (Finn et al., 2020) according to the following formulas: the low-CU alike model was constructed using the maximum score for each subject pair; the high-CU alike model was constructed using 1 minus the minimum score for each subject pair. The third nearest neighbors model was constructed using the absolute value of the difference between subject pairs’ scores. Prior to constructing each model, all subjects’ summary scores were normalized relative to the maximum CU score in the sample. To investigate finer-grained patterns of CU traits, we also constructed a nearest neighbors model by calculating the Euclidean distance between subject pairs’ item-wise responses on the ICU. Each matrix consisted of 435 unique combinations of subject pairs (30!/2! × (30 − 2)!).

**Figure 2.**
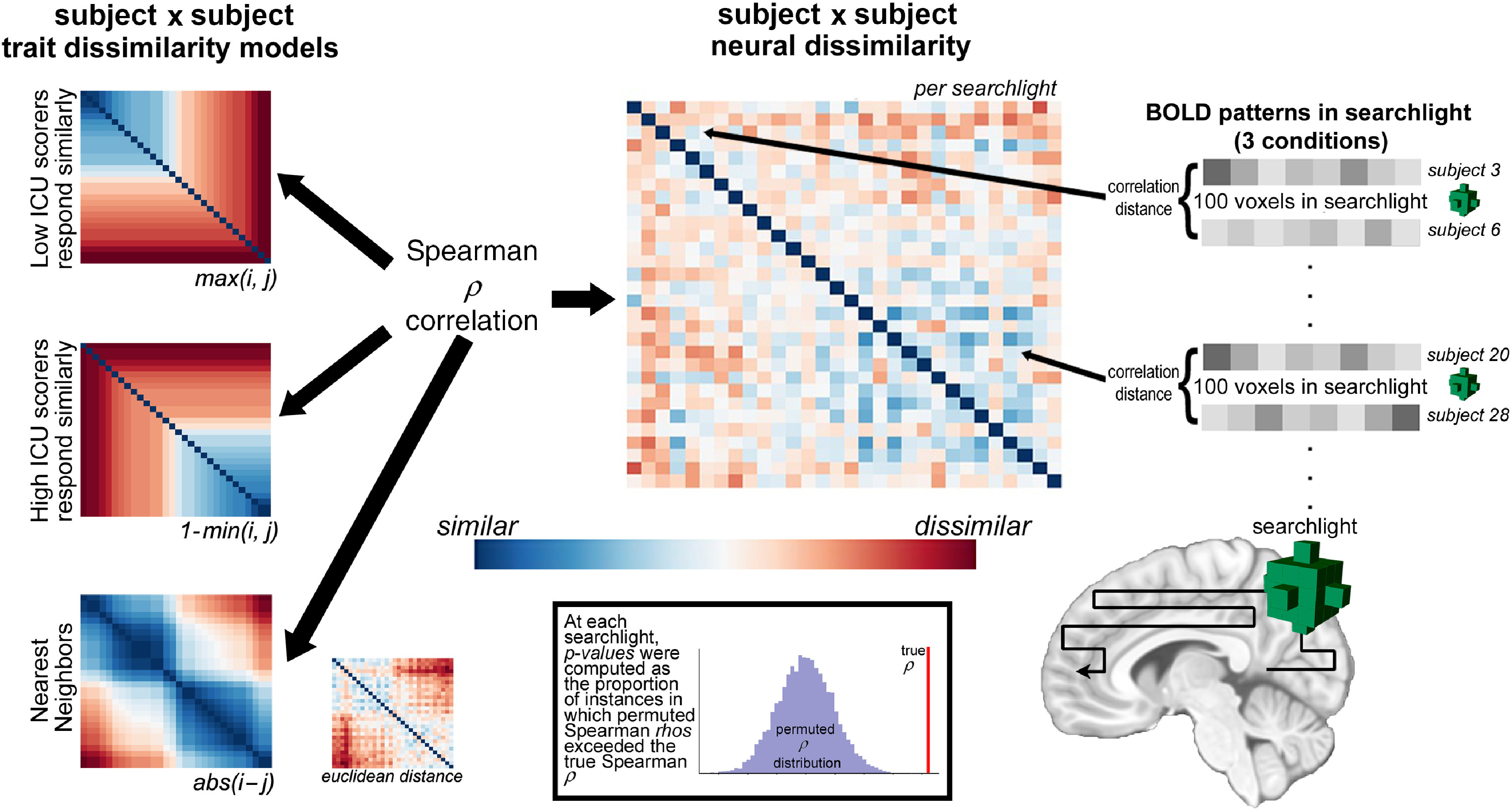
**Visualization of Searchlight Intersubject Representational Similarity Analysis.** Searchlight Intersubject Representational Similarity Analysis (IS-RSA) consisted of the following steps: (1) We computed three subject × subject disimilarity matrices based on CU summary scores across subjects. The first matrix tested a model in which low scoring CU adolescents’ neural response patterns were more alike while all others’ were different from each other; the second matrix tested a model in which high scoring CU adolescents’ neural response patterns were more alike while all others’ were different from each other; and the third matrix tested a model where adolescents’ neural response patterns were similar to each other in a relative rather than an absolute sense. Depicted trait dissimilarity models are sorted by ICU total scores in ascending order. (2) For each condition (“Afraid for you”, “Afraid for self”, “Afraid of you”), we then computed a subject × subject neural dissimilarity matrix within 100-voxel searchlights across gray matter. (3) Again for each condition, we vectorized the lower triangle of each matrix and performed a Spearman *ρ* correlation at each searchlight between intersubject behavioral dissimilarity and intersubject neural dissimilarity matrices, and assigned the *ρ* statistic to the center voxel in the searchlight. (4) Statistical significance was determined using a Mantel permutation test, in which both the rows and columns of the subject × subject model dissimilarity matrix were shuffled and the Spearman correlation between both correlation matrices was recomputed 1000 times to generate an empirical null distribution of rank correlations. (5) At each searchlight, we calculated the *p*-value as the proportion of instances in which the permuted Spearman *ρ* statistic exceeded the true Spearman *ρ* statistic.

Next, we searched for brain regions that responded to fearful facial expressions similarly 1) among high-CU subjects, 2) among low-CU subjects, and 3) for subjects who were similar in CU traits in a relative rather than an absolute sense. We conducted this analysis using a spherical searchlight comprised of 100 voxels. For each task condition (baseline, “afraid for you,” “afraid of you,” and “afraid for self”), we created a 30 × 30 dissimilarity matrix in each searchlight in terms of the multivoxel activity pattern correlation distances (1 minus Spearman *ρ*) between all pairs of participants while they viewed the expressions in each context (again, 435 unique combinations). We assessed correspondence between the lower triangles of these matrices using a Spearman *ρ* correlation and assigned *ρ* values to the center voxel of each searchlight. Statistical significance was determined using a Mantel permutation test (Mantel, 1967; Nummenmaa et al., 2012), in which both the rows and columns of one subject × subject dissimilarity matrix (e.g., subject labels) were shuffled and the Spearman correlation between both correlation matrices was recomputed 1000 times to generate an empirical null distribution of rank correlations (Figure 2). For each searchlight, we calculated the *p*-value as the proportion of instances in which the permuted Spearman *ρ* statistic exceeded the true Spearman *ρ* statistic, and thresholded the resulting maps using a cluster-forming threshold at *p* < .005 and cluster-extent threshold at *k* = 10, and using a false-discovery rate (FDR) at *q* = .05.

For each condition, we assessed the voxel-wise similarity between the nearest neighbors model constructed using subjects’ item-wise responses and the nearest neighbors model based on the absolute difference of subject pairs’ summary scores by conducting a Spearman rank correlation, list-wise excluding nonoverlapping voxels. Because these tests revealed significant similarity across voxels between models for all conditions (*ρ*_*baseline*_ = .78, *p* < .0001; *ρ*_*ForYou*_ = .76, *p* < .0001; *ρ*_*OfYou*_ = .68, *p* < .0001; *ρ*_*ForSelf*_ = .70, *p* < .0001), we opted to interpret results from the absolute difference nearest neighbors model across all conditions. We report findings using the Euclidean distance model in Table 2.

**Table 2.** Table of searchlight IS-RSA results meeting significance threshold.

Condition & Model	Brain Region(s)	Cluster Size	Spearman *ρ*	*P*-value	MNI Peak Coordinate		
***Baseline***					*x*	*y*	*z*
Low ICU Alike	Precuneus (R)	25	.257	<.0001^+^	9	−53	39
	Inferior Frontal Gyrus, pars opercularis (R)	55	−.166	<.0001^+^	50	14	9
High ICU Alike	Superior Temporal Gyrus, posterior division, extending to Superior Temporal Sulcus (R)	130	.277	<.0001^+^	69	−23	3
	Middle Temporal Gyrus, temporooccipital part (R)	130	.168	.001	69	−46	0
	Precentral Gyrus extending to Middle Cingulate Cortex (R)	62	.266	<.0001^+^	9	−20	51
	Precentral Gyrus to Middle Cingulate Cortex (L)	62	.163	.004	−10	−27	48
	Superior Frontal Gyrus (L)	15	.247	.001	−6	48	36
	Inferior Frontal Gyrus, pars opercularis (L)	31	.241	.002	−55	11	0
	Cerebral White Matter, extending to Superior Temporal Gyrus (L)	21	.227	<.0001^+^	−36	−12	−12
	Postcentral Gyrus, extending to Supramarginal Gyrus (R)	26	.177	.001	58	−20	21
	Middle Temporal Gyrus, temporooccipital part (R)	11	.171	.004	62	−57	−6
	Precuneus (L)	16	−.188	.001	−6	−65	21
ICU Nearest Neighbors (Absolute Difference)	Middle Temporal Gyrus, posterior division, extending to Superior Temporal Sulcus (R)	147	.409	<.0001^+^	69	−31	−12
	Planum Polare (L)	34	.229	<.0001^+^	−40	−8	−15
	Inferior Temporal Gyrus, posterior division (R)	47	.200	<.0001^+^	43	−23	−21
	Middle Temporal Gyrus, temporooccipital part (R)	11	.194	.003	54	−57	−3
	Cerebellum Lobule VI (R)	12	.188	.003	32	−57	−33
	Lateral Occipital Cortex, inferior division (R)	39	−.149	<.0001^+^	43	−68	−9
	Parahippocampal Gyrus, posterior division (L)	16	−.135	<.0001^+^	−14	−38	−12
	Lateral Occipital Cortex, superior division (R)	23	−.109	<.0001^+^	43	−83	30
ICU Nearest Neighbors (Euclidean Distance)	Middle Temporal Gyrus, posterior division, extending to Superior Temporal Sulcus (R)	117	.347	<.0001^+^	69	−31	−12
	Lingual Gyrus (L)	22	.229	.001	−10	−80	−6
	Superior Temporal Gyrus, anterior division (L)	30	.218	<.0001^+^	−51	−16	−15
	Lateral Occipital Cortex, superior division (R)	17	.213	<.0001^+^	20	−80	42
	Middle Occipital Gyrus (L)	12	.195	.002	−29	−76	18
	Middle Temporal Gyrus, temporooccipital part (R)	21	.179	.001	54	−57	−3
	Parahippocampal Gyrus, anterior division (R)	11	.170	.001	32	−23	−24
***Afraid for You***							
Low ICU Alike	Intracalcarine Cortex (R)	43	.436	<.0001^+^	5	−80	6
	Dorsolateral Prefrontal Cortex (R)	41	.334	<.0001^+^	32	44	39
	Frontal Operculum Cortex (R)	11	.292	.001	43	22	3
	Medial Prefrontal Cortex (L, R)	16	.289	.004	−6	29	−18
	Middle Temporal Gyrus, posterior division (R)	23	.269	<.0001^+^	65	−12	−21
	Paracingulate Gyrus, extending to Anterior Cingulate Cortex (L, R)	30	.263	.002	2	48	3
	Precuneus (R)	204	−.325	<.0001^+^	17	−72	36
	Lateral Occipital Cortex, superior division (R)	204	−.220	.002	50	−61	48
	Superior Parietal Lobule (R)	204	−.213	.001	28	−57	54
	Precentral Gyrus, lateral part (R)	97	−.265	.001	43	−5	63
	Middle Frontal Gyrus (R)	97	−.184	.002	50	11	39
	Hippocampus (R)	23	−.238	.003	39	−31	−9
	Lateral Occipital Cortex, superior division	19	−.174	.001	−25	−68	63
High ICU Alike	Occipital Fusiform Gyrus (R)	39	.551	<.0001^+^	24	−80	−9
	Cerebellum Lobule VI (R)	39	.242	<.0001^+^	17	−65	−21
	Precuneus (R)	109	.498	<.0001^+^	20	−72	33
	Lateral Occipital Cortex, superior division (L)	109	.230	.003	−6	−83	48
	Occipital Fusiform Gyrus (R)	14	.407	.005	35	−38	−18
	Supramarginal Gyrus, posterior division (R)	128	.399	<.0001^+^	62	−38	36
	Supramarginal Gyrus, anterior division (R)	128	.310	<.0001^+^	69	−20	39
	Lateral Occipital Cortex, superior division (L)	18	.342	.001	−17	−80	36
	Superior Parietal Lobule (R)	15	.273	<.0001^+^	17	−53	57
	Inferior Frontal Gyrus, pars opercularis (R)	42	.270	<.0001^+^	54	22	24
	Middle Frontal Gyrus (R)	29	.261	.001	47	7	54
	Postcentral Gyrus, lateral part (L)	15	−.247	.003	−55	−12	33
	Middle Frontal Gyrus (R)	21	−.226	<.0001^+^	35	26	36
ICU Nearest Neighbors (Absolute Difference)	Inferior Frontal Gyrus, pars opercularis (R)	54	.283	.001	62	18	6
	Supramarginal Gyrus, Temporoparietal Junction (R)	114	.262	<.0001^+^	69	−27	42
	Occipital Fusiform Gyrus (R)	32	.234	.001	17	−76	−18
	Temporal Occipital Fusiform Cortex (R)	30	.225	<.0001^+^	32	−46	−18
	Superior Temporal Gyrus, anterior division (R)	13	.222	.002	58	−1	−6
	Anterior Cingulate Cortex (R)	31	.220	.001	2	41	3
	Intracalcarine Cortex (R)	17	.214	<.0001^+^	2	−80	6
	Supramarginal Gyrus, posterior division (L)	33	.206	.001	−55	−42	27
	Inferior Frontal Gyrus, pars opercularis (R)	13	.188	.001	54	22	24
	Central Opercular Cortex (L)	11	−.135	<.0001^+^	−47	−16	12
	Superior Parietal Lobule (L)	11	−.122	<.0001^+^	−36	−53	51
ICU Nearest Neighbors (Euclidean Distance)	Temporal Occipital Fusiform Cortex (R)	124	.310	<.0001^+^	32	−46	−18
	Cerebellum Lobule VI (R)	124	.219	<.0001^+^	13	−61	−24
	Cerebellum Lobule IV and V (L)	124	.180	.003	−2	−53	−12
	Intracalcarine Cortex (L)	77	.291	.001	−10	−83	0
	Occipital Fusiform Gyrus (L)	56	.275	<.0001^+^	−32	−80	−21
	Occipital Fusiform Gyrus (R)	14	.236	.003	20	−87	−18
	Middle Occipital Gyrus (L)	11	.231	<.0001^+^	−21	−95	3
	Frontal Operculum Cortex (R)	30	.225	.001	47	18	0
	Supramarginal Gyrus, anterior division (R)	43	.221	.001	69	−23	42
	Dorsolateral Prefrontal Cortex (R)	11	.214	<.0001^+^	20	56	27
	Dorsolateral Prefrontal Cortex (R)	11	.175	.002	28	48	39
	Anterior Cingulate Cortex (R)	11	.167	.003	2	44	3
	Postcentral Gyrus, dorsomedial part (R)	14	−.155	.001	2	−38	69
	Posterior Cingulate Cortex (R)	20	−.144	<.0001^+^	5	−31	42
***Afraid of You***							
Low ICU Alike	Intracalcarine Cortex (R)	18	.368	.002	5	−83	6
	Posterior Cingulate Cortex (R)	40	−.328	<.0001^+^	5	−31	30
	Hippocampus (L)	41	−.316	<.0001^+^	−32	−31	−6
	Lingual Gyrus (L)	41	−.105	.002	−25	−53	−3
	Cuneal Cortex (R)	19	−.285	.001	17	−72	30
	Superior Parietal Lobule	16	−.279	<.0001^+^	−17	−53	63
	Middle Temporal Gyrus, posterior division (R)	45	−.278	<.0001^+^	47	−20	−9
	Lateral Occipital Cortex, superior division (R)	16	−.252	.001	35	−80	42
	Lateral Occipital Cortex, superior division (R)	17	−.226	.003	39	−80	12
	Postcentral Gyrus, dorsomedial part (R)	19	−.217	<.0001^+^	13	−42	66
	Middle Temporal Gyrus, temporooccipital part (R)	11	−.208	.002	62	−53	3
	Lateral Occipital Cortex, superior division (L)	31	−.184	.003	−55	−72	30
	Dorsolateral Prefrontal Cortex (L)	12	−.144	.002	−17	59	33
High ICU Alike	Posterior Cingulate Cortex (L)	29	.411	.002	5	−27	33
	Intracalcarine Cortex (L)	23	−.271	.002	−6	−76	6
ICU Nearest Neighbors (Absolute Difference)	Lateral Occipital Cortex, superior division (R)	28	−.152	<.0001^+^	32	−83	39
	Lateral Occipital Cortex, superior division (L)	32	−.151	<.0001^+^	−21	−61	54
	Middle Frontal Gyrus (R)	20	−.138	.001	43	11	30
	Paracingulate Gyrus (R)	20	−.132	.002	9	22	45
	Brain-Stem (L)	34	−.120	<.0001^+^	−10	−46	−45
	Dorsolateral Prefrontal Cortex (R)	18	−.117	.001	35	56	27
	Lateral Occipital Cortex, inferior division (R)	17	−.111	.003	43	−72	15
	Lateral Occipital Cortex, superior division (R)	28	−.152	<.0001^+^	32	−83	39
ICU Nearest Neighbors (Euclidean Distance)	Intracalcarine Cortex (L)	52	.264	.001	−6	−80	3
	Frontal Operculum Cortex (L)	12	.245	<.0001^+^	−44	26	0
	Middle Occipital Gyrus (L)	14	.244	<.0001^+^	−21	−95	3
	Cerebellum Lobule VI (R)	37	.237	.001	17	−65	−27
	Superior Temporal Gyrus, anterior division (L)	13	.210	.001	−51	−12	−9
	Lateral Occipital Cortex, superior division (L)	18	−.170	<.0001^+^	−10	−61	69
	Middle Temporal Gyrus, temporooccipital part (R)	43	−.170	.002	65	−57	6
	Lateral Occipital Cortex, inferior division (R)	43	−.162	<.0001^+^	54	−76	12
	Middle Temporal Gyrus, temporooccipital part (R)	43	−.141	.001	43	−57	12
***Afraid for Self***							
Low ICU Alike	Caudate (R)	12	.174	.001	17	14	18
	Insular Cortex (L)	53	−.220	<.0001^+^	−36	11	−6
	Putamen (L)	53	−.172	<.0001^+^	−29	−12	6
	Cerebellum Lobule VI (L)	15	−.206	.001	−17	−68	−24
	Superior Parietal Lobule (R)	20	−.190	.003	28	−53	48
	Precuneus (R)	13	−.185	.003	13	−38	57
	Precentral Gyrus, lateral part (L)	17	−.180	.003	−29	−12	57
	Postcentral Gyrus, dorsomedial part (L)	13	−.166	.004	−17	−38	45
High ICU Alike	Central Opercular Cortex (L)	20	−.284	<.0001^+^	−51	−5	9
	Superior Frontal Gyrus (R)	26	−.217	<.0001^+^	2	14	63
	Lateral Occipital Cortex, superior division (R)	11	−.212	<.0001^+^	28	−83	39
	Cerebellum Crus II (R)	13	−.173	.003	13	−80	−33
	Entorhinal cortex, anterior division (L)	18	−.153	<.0001^+^	−25	3	−15
ICU Nearest Neighbors (Absolute Difference)	Inferior Frontal Gyrus, pars triangularis (R)	16	.240	<.0001^+^	50	18	−3
	Angular Gyrus (R)	44	.233	<.0001^+^	54	−50	18
	Frontal Pole extending to Dorsolateral Prefrontal Cortex (R)	11	.185	.001	17	63	15
	Precentral Gyrus, lateral part (R)	113	−.172	<.0001^+^	43	−12	36
	Postcentral Gyrus, lateral part (R)	113	−.137	<.0001^+^	62	−16	30
	Postcentral Gyrus, lateral part (L)	10	−.142	.001	−44	−16	33
	Precentral Gyrus, lateral part (L)	29	−.142	<.0001^+^	−32	−8	45
	Brain-Stem (R)	27	−.141	.002	13	−38	−21
	Supramarginal Gyrus, posterior division (L)	11	−.137	<.0001^+^	−51	−42	33
	Frontal Pole extending to Ventrolateral Prefrontal Cortex (R)	12	−.125	.001	47	44	−15
ICU Nearest Neighbors (Euclidean Distance)	Inferior Frontal Gyrus, pars triangularis (R)	16	.242	.001	50	18	−3
	Occipital Pole (L)	12	.222	<.0001^+^	−17	−102	6
	Frontal Pole extending to Dorsolateral Prefrontal Cortex (R)	16	.217	.001	24	63	15
	Precuneus (L)	11	.194	.001	−2	−68	48

***Note.*** Results are reported using a cluster-forming threshold at *p* < .005 and cluster-extent threshold at *k* = 10 with voxel size = 3mm^3^. ^+^ indicates a cluster surviving false discovery rate (FDR) correction at *q* = .05. *ρ* values represent the degree to which the specific cluster corresponds to the inter-subject model with positive values indicating higher correspondence and negative values indicating anticorrespondence. Regions were labeled using the Harvard Oxford Atlas, and organized by condition, intersubject model, positive-to-negative *ρ* values, and then posterior-to-anterior.

## 2. Results

### 2.1 Behavioral data

Age was neither related to ICU total nor any of the subscales (*rs* < .16, *ps* > .4). Gender was related to CU traits with males (*M* = 40.31, *SE* = 3.00) scoring higher than females (*M* = 30.79, *SE* = 3.75), *t*(28) = 2.00, *p* = .03. Males also had higher scores than females on the unemotional (*t*(28) = 1.70, *p* = .049) and uncaring (*t*(28) = 2.05, *p* = .025) subscales, and their scores trended higher on the callous (*t*(28) = 1.64, *p* = .056).

Behavioral data during the fMRI task consisted of adolescents’ responses to our attention-check questions in the final four runs of the fMRI task, where they pressed one of three buttons to confirm that they read and understood each prompt. The average accuracy score was 85.83% (*SD* = 2.72). A Poisson regression analysis controlling for age, gender, and IQ indicated no statistically significant association between number of errors and CU traits (ICU total) (*β* = .009, *SE* = .008, *p* = .29) or any covariates (.18 < *p* < .51).

### 2.2 Neuroimaging

#### 2.2.1 Univariate

Our first analysis assessed how activation in response to fearful facial expressions varied as a function of CU traits using a traditional univariate approach across the whole-brain and within anatomically defined *a priori* bilateral amygdala masks. Multiple regression analyses revealed no statistically significant association between CU traits (total, callousness, uncaring, or unemotional) and neural activation to fearful faces at baseline when examining the whole brain. We found a significant main effect of activation to fearful faces versus fixation among all subjects in a small cluster in left visual cortex (13 voxels). Simultaneously, another multiple regression model predicting neural activation to fearful facial expressions across the different interpretative contexts found neither a significant effect of context nor an effect of CU traits. Applying small-volume correction in bilateral amygdala did not yield any findings across these two models.

Only when averaging across voxels in right amygdala did we find negative associations between mean activation and CU traits, controlling for age, gender, and IQ. During the “Afraid for Self” condition, we found associations between mean right amygdala activity and callousness (*partial ρ* = −.409, *p* = .02) and ICU total scores (*partial ρ* = −.349, *p* = .06). We present plots of mean activation as a function of CU traits for each context in bilateral, right, and left amygdala in the Supplementary Materials.

#### 2.2.2 Intersubject RSA

We next investigated whether multivoxel neural activation patterns associated with viewing fearful facial expressions in the different contexts would correspond to each of our three models (Table 2). Our first analyses focused on the baseline runs, during which the context was unspecified. Here, results from the IS-RSA revealed that a cluster in the precuneus (*ρ* = .257, *p* < .0001) exhibited an intersubject representational geometry where low-CU adolescents’ neural response patterns were similar while others’ were dissimilar during baseline fearful face viewing. A much larger set of clusters exhibited neural response patterns whereby high-CU adolescents’ neural response patterns were similar while others’ were dissimilar, including right posterior superior temporal gyrus extending to posterior superior temporal sulcus (pSTS) (*ρ* = .277, *p* < .0001), right middle temporal gyrus (MTG) (*ρ* = .168, *p* = .001), bilateral precentral gyrus (*ρ* = .266, *p* < .0001), left superior frontal gyrus (*ρ* = .247, *p* = .001), pars opercularis portion of the left inferior frontal gyrus (IFG) (*ρ* = .241, *p* = .002), left anterior STG (*ρ* = .227, *p* < .0001), and right postcentral gyrus extending to supramarginal gyrus (*ρ* = .177, *p* = .001). Finally, adolescents who are similar in CU traits, regardless of whether they were low or high, exhibited similar neural responses in regions that included right posterior MTG (pMTG) extending to pSTS (*ρ* = .409, *p* < .0001), right posterior inferior temporal gyrus (*ρ* = .200, *p* < .0001), and right MTG (*ρ* = .194, *p* = .003).

Our next analyses focused on response patterns during the cued contexts. When expressers were described as afraid for the viewer (“Afraid for you” condition), results from the IS-RSA revealed that specific regions exhibited an intersubject representational geometry where low-CU adolescents’ neural response patterns are similar while others’ are dissimilar. Identified brain regions included right intracalcarine cortex (*ρ* = .436, *p* < .0001), right frontal pole (*ρ* = .334, *p* < .0001), right frontal operculum (*ρ* = .292, *p* = .001), bilateral medial prefrontal cortex (*ρ* = .289, *p* = .004), right pMTG (*ρ* = .269, *p* < .0001), and bilateral paracingulate gyrus, extending to anterior cingulate cortex (ACC) (*ρ* = .263, *p* = .002). Results in this condition also showed geometry whereby high-CU adolescents’ neural response patterns were similar while others’ were dissimilar in regions including several early-visual stream areas: bilateral superior occipital cortex (*ρ*_*left*_ = .230, *p* = .003; *ρ*_*right*_ = .342, *p* = .001) and two clusters in right FFG (*ρ* = .551, *p* < .0001; *ρ* = .407, *p* = .005); and right cerebellum lobule VI (*ρ* = .242, *p* < .0001), right precuneus (*ρ* = .498, *p* < .0001), right posterior supramarginal gyrus (*ρ* = .399, *p* < .0001), right anterior supramarginal gyrus (*ρ* = .310, *p* < .0001), right superior parietal lobule (*ρ* = .273, *p* < .0001), pars opercularis portion of the right IFG (*ρ* = .270, *p* < .0001), and right middle frontal gyrus (*ρ* = .261, *p* = .001). Finally, among adolescents who were similar in CU traits, regardless of whether they were high or low on the scale, similar neural response patterns were observed in two clusters in the pars opercularis portion of right IFG (*ρ* = .283, *p* = .001; *ρ* = .188, *p* = .001), right temporoparietal junction (*ρ* = .262, *p* < .0001), two clusters in right FFG (*ρ* = .234, *p* = .001; *ρ* = .225, *p* < .0001), right aSTG (*ρ* = .222, *p* = .002), right ACC (*ρ* = .220, *p* = .001), right intracalcarine cortex (*ρ* = .214, *p* < .0001), and left posterior supramarginal gyrus (*ρ* = .206, *p* = .001)

When expressers were described as afraid of the viewer (“Afraid of you” condition), IS-RSA results revealed that low-CU adolescents’ neural response patterns were similar while others’ were dissimilar in right intracalcarine cortex (*ρ* = .368, *p* = .002). High-CU adolescents’ exhibited similar neural response patterns while others’ were dissimilar in left posterior cingulate cortex (PCC) (*ρ* = .411, *p* = .002). We did not identify any regions that corresponded to the nearest neighbor model in this condition.

In the “Afraid for self” condition (i.e., when expressers were described as afraid for themselves), right caudate was the only cluster that corresponded to the low-CU alike model (*ρ* = .174, *p* = .001). We did not identify any clusters meeting significance threshold that corresponded to the high-CU alike model. The only clusters that corresponded to the nearest neighbor model were the pars triangularis portion of the right IFG (*ρ* = .240, *p* < .0001), right angular gyrus (*ρ* = .233, *p* < .0001), and right frontal pole (*ρ* = .185, *p* = .001).

**Figure 3.**
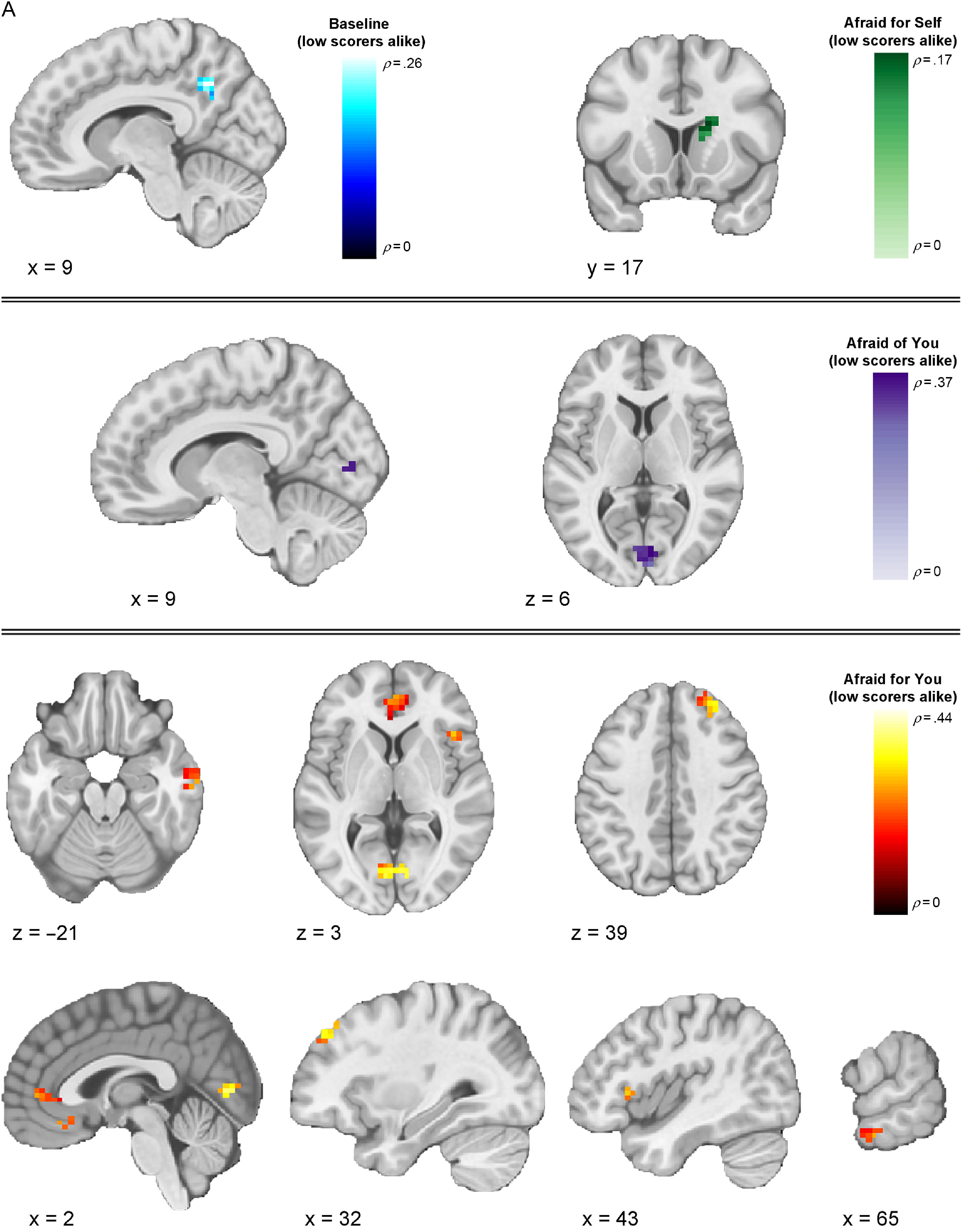
**Thresholded IS-RSA Results (Low CU Scorers Alike Model).** Visualization of clusters across conditions showing significant intersubject pattern response structure whereby low-CU adolescents were similar while all others were dissimilar. Clusters are thresholded at *p* < .005 and *k* = 10 (FDR-corrected results are reported in Table 2).

**Figure 4.**
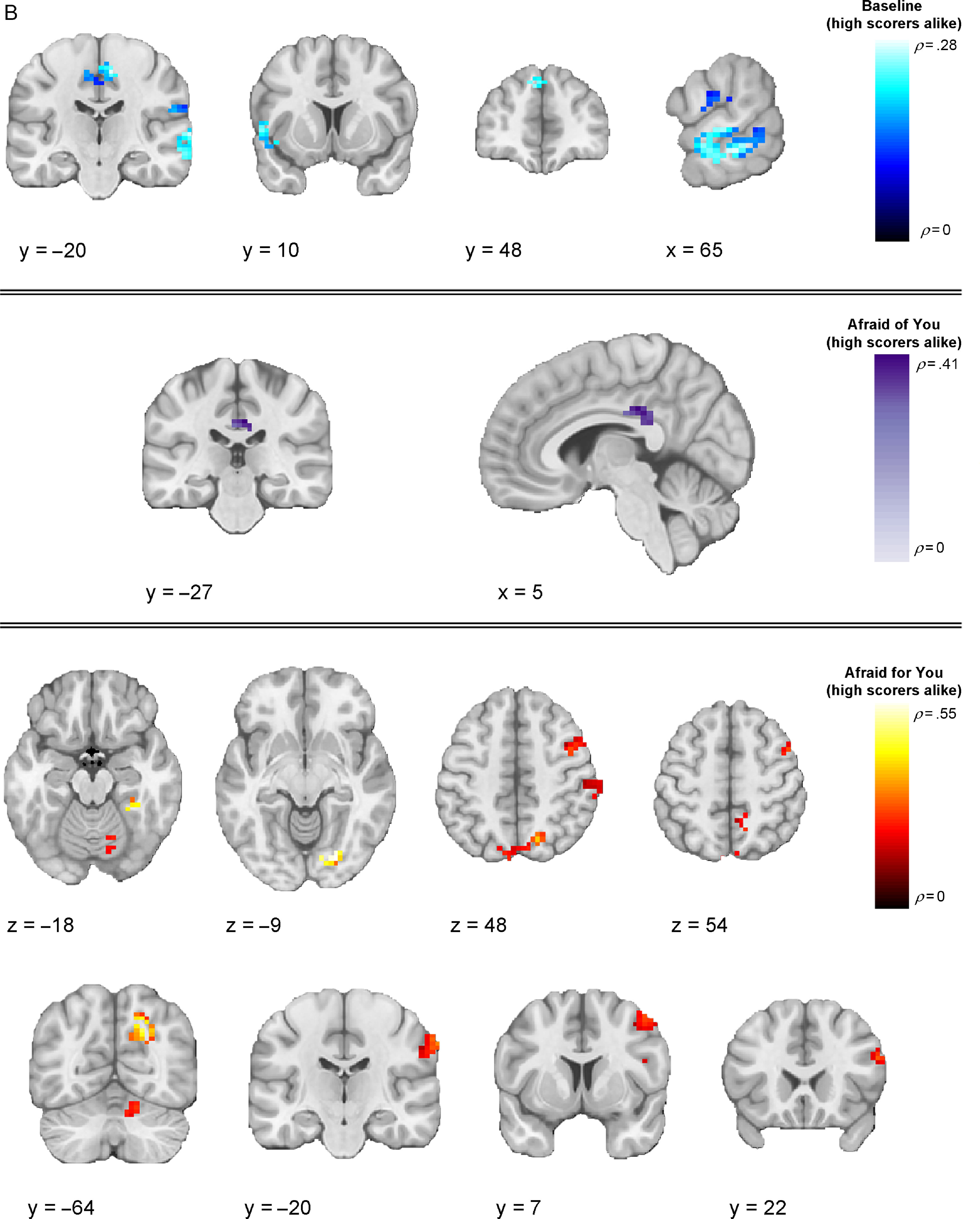
**Thresholded IS-RSA Results (High CU Scorers Alike).** Visualization of clusters across conditions showing significant intersubject pattern response structure whereby high-CU adolescents were similar while all others were dissimilar. Clusters are thresholded at *p* < .005 and *k* = 10 (FDR-corrected results are reported in Table 2).

**Figure 5.**
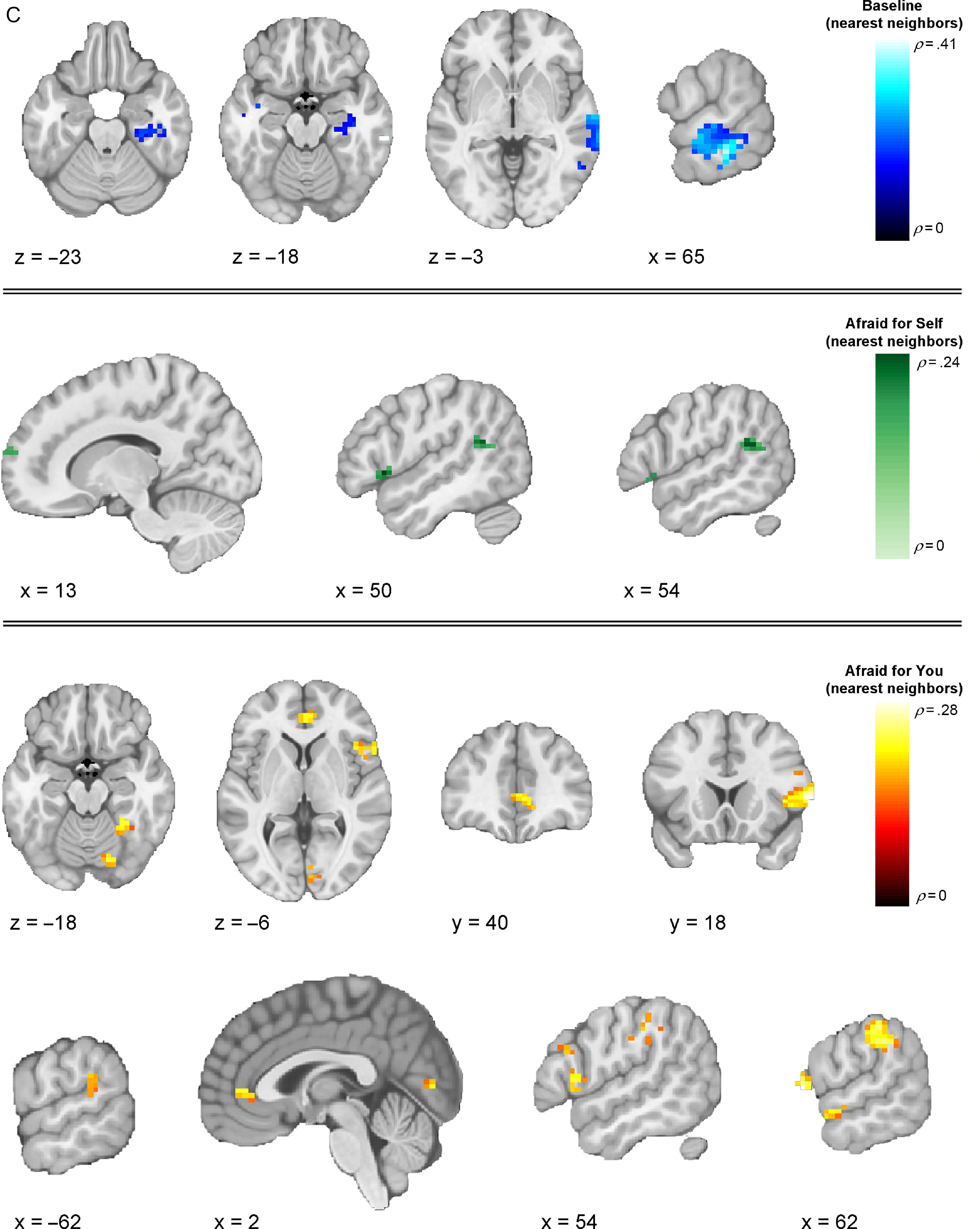
**Thresholded IS-RSA Results (Nearest Neighbors Model).** Visualization of clusters across conditions showing significant intersubject pattern response structure whereby adolescents were similar in CU traits regardless of being low or high. Clusters are thresholded at *p* < .005 and *k* = 10 (FDR-corrected results are reported in Table 2).

Regions showing a negative relationship with any of the models (clusters showing structure that is anticorrelated with the model) are listed in Table 2, but (for clarity) only clusters showing a positive relationship across models are displayed in Figures 3–5. Thresholded and unthresholded group-level statistical maps for IS-RSA are available in Supplementary Materials.

## 3. Discussion

The present findings provide the first evidence that context-specific patterns of brain activity in response to fearful facial expressions align when people occupy similar positions in a phenotypic feature space of CU traits. IS-RSA identified how variations in adolescent- and parent-reported CU traits directly map onto variations in neural activation patterns associated with the context-specific interpretation of fearful facial expressions. Of note, we generally found activation patterns in regions implicated in low-level visual processing (e.g., occipital cortex), and the detection of personal threat from social cues (e.g., ACC) exhibited intersubject structure whereby low-CU adolescents were alike and others were dissimilar. By contrast, activation patterns in regions implicated in emotional face processing and perception (e.g., FFG, STG, pSTS) and higher-level social cognition (e.g., lPFC, mPFC, PCC, precuneus) more often showed intersubject structure whereby high-CU adolescents were alike while others were more dissimilar. These findings were highly right-lateralized, consistent with evidence for the right lateralization of emotional face processing and related socioaffective processes (Gläscher & Adolphs, 2003; Hung et al., 2010; Noesselt, Driver, Heinze, & Dolan, 2005). CU traits also shaped patterns of responses to fearful expressions as a function of interpretative contexts, providing insight beyond prior findings of reduced amygdala responsiveness to these expressions (Byrd, Kahn, & Pardini, 2013; Cardinale & Marsh, 2017; Carré et al., 2013; Ciucci, Baroncelli, Franchi, Golmaryami, & Frick, 2014; Essau, Sasagawa, & Frick, 2006; Houghton, Hunter, & Crow, 2013; Marsh et al., 2008) – a finding that was partially replicated here, with CU traits linked to reduced univariate amygdala responsiveness to fearful expressions only when expressers were described as afraid for themselves.

In line with evidence from our univariate findings regarding the importance of contextual framing, our IS-RSA analyses for the first-time link intersubject similarities in neural response patterns to contextualized fearful expressions to differential intersubject models of CU traits. Two superordinate novel patterns emerged when youths varying in CU traits viewed fearful facial expressions across varying contexts. First, neural response patterns among low-CU adolescents became more idiosyncratic as emotional face information moved along the cortical processing hierarchy (as evidenced by the high-CU alike model). By contrast, neural response patterns aligned more among high-CU adolescents in regions along the entire cortical hierarchy, especially those that are implicated in social information processing, such as the FFG, STG, pSTS, PCC, and lPFC. These patterns may reflect these youths’ impairments in interpreting and responding to the social messages that fearful facial expressions convey. Second, these observations vary somewhat according to the context in which the fearful facial expressions are interpreted. Specifically, higher-order association regions exhibit intersubject neural activity pattern alignment even among low-CU adolescents when the context is specified. These findings align with recent work demonstrating the importance of contextual information for understanding emotional faces (Davis, Neta, Kim, Moran, & Whalen, 2016; Petro, Tong, Henley, & Neta, 2018).

Using IS-RSA to investigate neural responding to fearful expressions, we found that intersubject neural response patterns reflected intersubject differences in CU traits, especially in contexts where the expressers were described as afraid for the participant (“Afraid for you” condition). For example, we found higher alignment for high-CU participants in right FFA (while other participants show more idiosyncratic patterns). Known for its role in face identity processing (Kanwisher, 2017; Kanwisher, McDermott, & Chun, 1997), the right FFA also plays a role in emotion category discrimination, with selectivity for fearful facial expressions possibly resulting from feedback from the amygdala and mPFC, as suggested by findings from human fMRI and intracranial recordings (Harry, Williams, Davis, & Kim, 2013; Ishai, Pessoa, Bikle, & Ungerleider, 2004; Kawasaki et al., 2012). The observed pattern alignment among multivoxel patterns in this region may underpin impairments in the category-based perceptions of fearful facial expressions in CU adolescents, potentially from disrupted modulation from the amygdala.

We also observed differential alignment across high- and low-CU adolescents in regions implicated in emotional appraisal and introspection, as well as defensive responses to threats. Also within the “Afraid for you” context, low-CU adolescents were more similar (and other adolescents were most dissimilar to each other) in regions that included mPFC and ACC. Previous studies have found that perspective-taking, emotional appraisal, and cognitive empathy tasks recruit mPFC in both adults (Bzdok et al., 2012; Rameson, Morelli, & Lieberman, 2012; Schurz, Radua, Aichhorn, Richlan, & Perner, 2014; Tusche, Bockler, Kanske, Trautwein, & Singer, 2016; Van Overwalle, 2009) and adolescents (Sebastian et al., 2012). For example, mPFC activation increases when adolescents perform tasks that require making inferences about social interactions (Kilford, Garrett, & Blakemore, 2016; Sebastian, Burnett, & Blakemore, 2008; Vollm et al., 2006). mPFC is also functionally correlated with the amygdala during fearful face processing, which is compromised in CU individuals (Blair, 2008; Breeden, Cardinale, Lozier, VanMeter, & Marsh, 2015; Marsh et al., 2008). Evidence also demonstrates that ACC activates in response to threat-related stimuli (Bishop, Duncan, Brett, & Lawrence, 2004), exhibits strong structural and functional connectivity with the amygdalae (Carlson, Cha, & Mujica-Parodi, 2013; Kim et al., 2011; Williams et al., 2006), and displays blunted responses to others’ distress in CU youths (Lockwood et al., 2013). Together, the present findings suggest a deficit within these regions among high-CU adolescents, particularly when viewing social cues that are contextualized as cooperative social signals.

Intersubject differences among subjects viewing fearful faces in the “Afraid of you” and “Afraid for self” conditions were less sensitive to the models testing relative and absolute differences among subjects. This could suggest that univariate patterns of activation better explain variation among CU adolescents within these contexts (particularly as we observed the stereotypical association between CU traits and reduced univariate amygdala response in the “afraid for self” condition). Notably, when expressers were described as afraid for themselves, we observed that low-CU adolescents were more similar (and all others dissimilar) in right caudate. Individual differences in activation to fearful facial expressions within this region has been linked to CU traits (Lozier et al., 2014), in which increased activation was related to lower CU traits. This finding in particular may reflect this region’s role in motivating prosocial approach towards relieving another’s personal distress (Schlund, Magee, & Hudgins, 2011).

### 3.1 Limitations

The reported findings should be considered in light of several limitations. First, our task was not selected for its ecological validity, but rather because the incorporated prompts allowed us to specify the context in which each expression should be interpreted. In the real world, of course, perceivers typically interpret others’ facial expressions using a larger variety of rich contextual cues and semantic information that require shifts in attention and reference to prior knowledge (Becker, 2009). Future studies should explore how more naturalistic cues related to the context of emotional facial expressions shape responses to and interpretations of these expressions.

Our MRI session included six runs of passively viewed fearful facial expressions, such that attentional requirements of the task should be considered. While the initial two baseline runs oriented and habituated subjects to the fearful expressions, and attentional checks were included in the final four runs, it is possible that neural responses related to attention could vary as a function of CU traits. Mitigating this concern, however, is that we did not find a relationship between CU traits and accuracy in the attention checks. Our sample size also was limited by practical considerations and our goal of oversampling adolescents with moderate- to high- CU traits.

It is also important to note that greater similarity in neural activity patterns does not reflect increased or reduced activity. High similarity could occur if two participants exhibit different mean levels of activation in a given brain region if the participants covary in their multivoxel activity patterns similarly. Additionally, our study utilizes anatomically-aligned data across pairs of subjects, but evidence suggests that functional alignment (e.g., hyperalignment) can improve detection of individual differences (Feilong, Nastase, Guntupalli, & Haxby, 2018). Our findings warrant further investigations to examine interindividual neural responses to the contextualization of fearful facial expressions in association with CU traits and other psychological constructs – particularly given the novelty of the approach.

It should lastly be noted that although CU traits are often understood to be a unitary construct, recent studies found that ICU subcomponents (which include uncaring, callousness, and unemotionality) exhibit divergent associations with external psychological and neural variables (Cardinale et al., 2018; Cardinale & Marsh, 2017). In light of this, we calculated intersubject models for the ICU subscales separately and present these intersubject models in Supplementary Materials for comparison to the models constructed using ICU total scores.

## 4. Conclusions

Prior studies have consistently indicated disrupted neural responses to fearful facial expressions in CU youths, and found evidence that these disruptions are closely linked to the disruptive behavior characteristic of CU traits. Nonetheless, many questions remain regarding the nature of the observed disruptions. While some of these questions reflect the standard approaches to assessing responses to fearful expressions in CU youths, the present study observed more specific information about how CU traits disrupt responding to fearful expressions both by constraining the context in which the expressions were interpreted and applying a novel analytic approach that yields greater specificity in mapping CU traits onto patterns of neural responses. This work underscores the utility of using techniques that explore interindividual variation in behavioral and personality characteristics and multivoxel patterns of neural responding.

## Data deposit

Data and code are available on the Open Science Framework at https://osf.io/bqdm5.

## Supplementary material

For supplementary material accompanying this paper visit https://doi.org/10.1017/pen.2020.13. click here to view supplementary material
