# Supplementary material for: Mapping neural activity patterns to contextualized fearful facial expressions onto callous-unemotional (CU) traits: intersubject representational similarity analysis reveals less variation among high-CU adolescents
Source: Personal Neurosci. 2020 Nov 10;3:e12. doi: 10.1017/pen.2020.13 (PMC7681174; doi:10.1017/pen.2020.13)
Supplement: Supplementary file 1 [file S2513988620000139sup001.pdf]

Supplementary Figures 1A-H. Scatterplots of mean amygdala activation to fearful faces and CU traits across conditions using the Harvard Oxford Parcellation (baseline: blue; afraid for self: green; afraid for you: red-orange; afraid of you: purple)

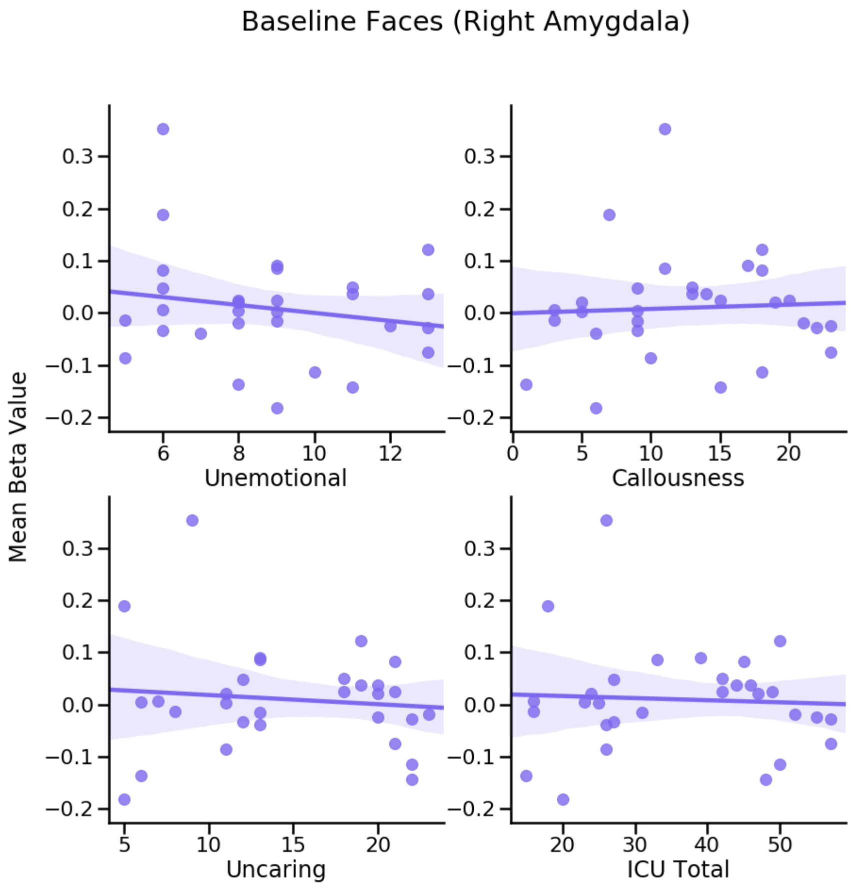

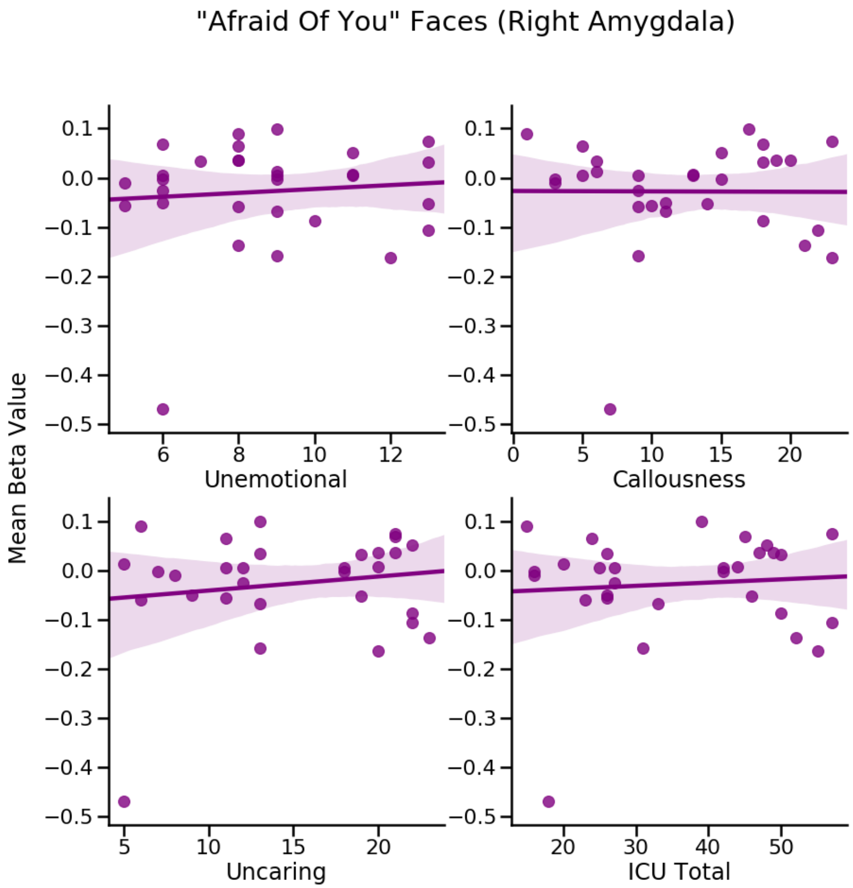

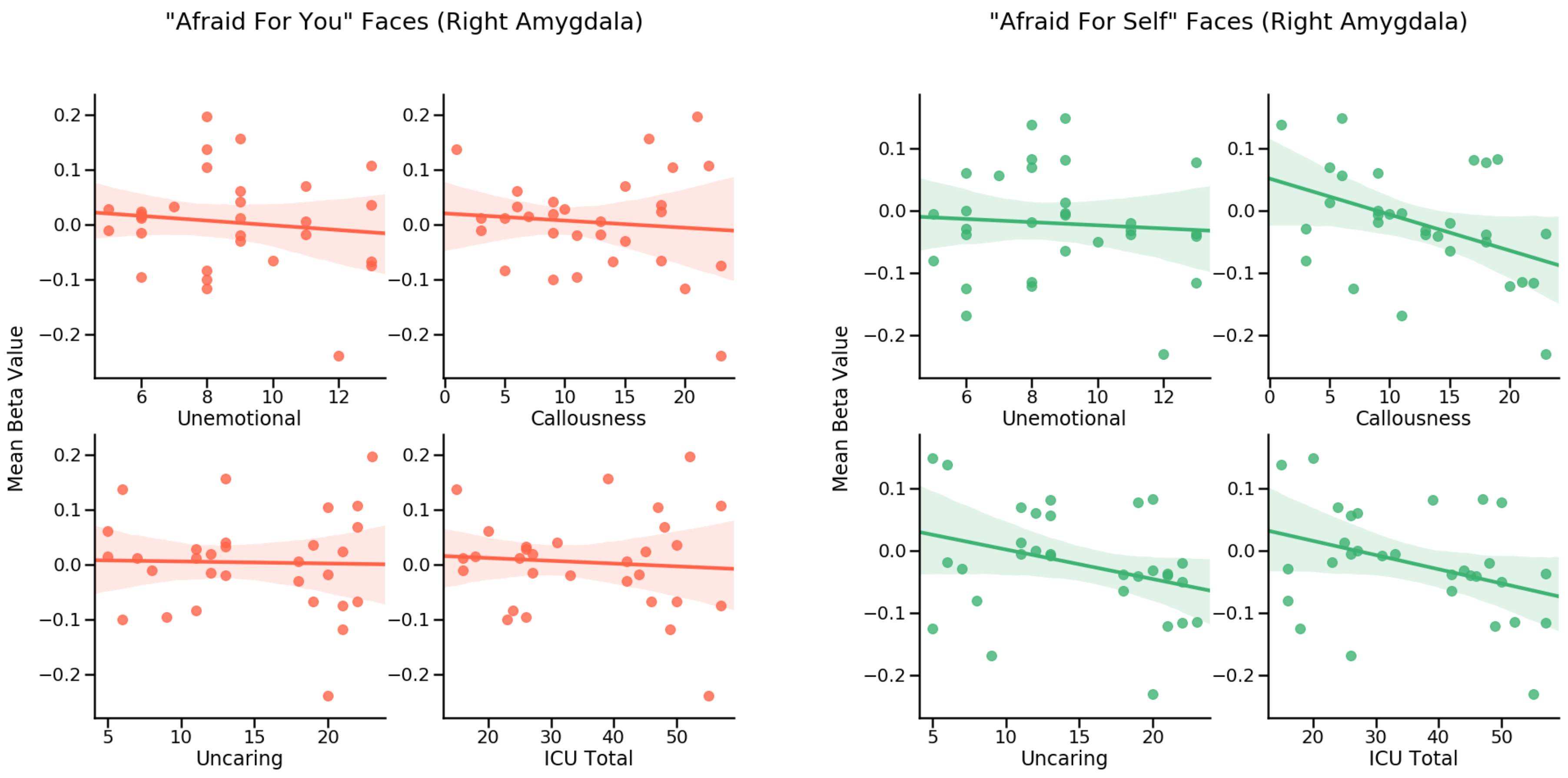

"Afraid For You" Faces (Right Amygdala)
"Afraid For Self" Faces (Right Amygdala)
Mean Beta Value
Unemotional
Callousness
Uncaring
ICU Total
Mean Beta Value
Unemotional
Callousness
Uncaring
ICU Total

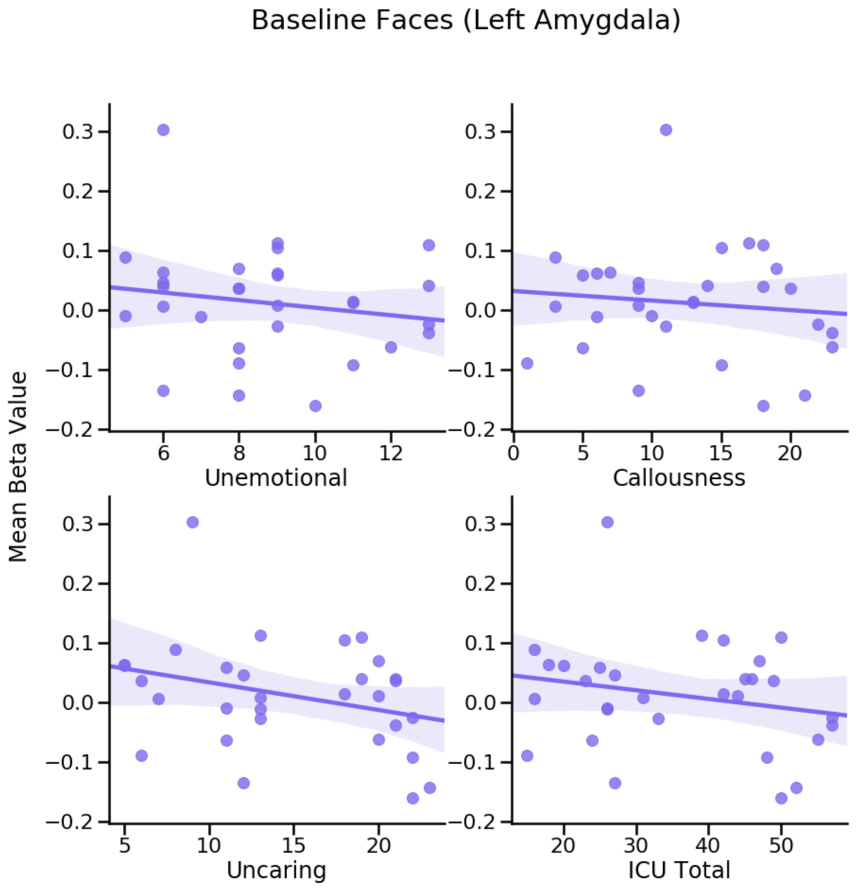
Baseline Faces (Left Amygdala)
Mean Beta Value
Unemotional
Callousness
Uncaring
ICU Total

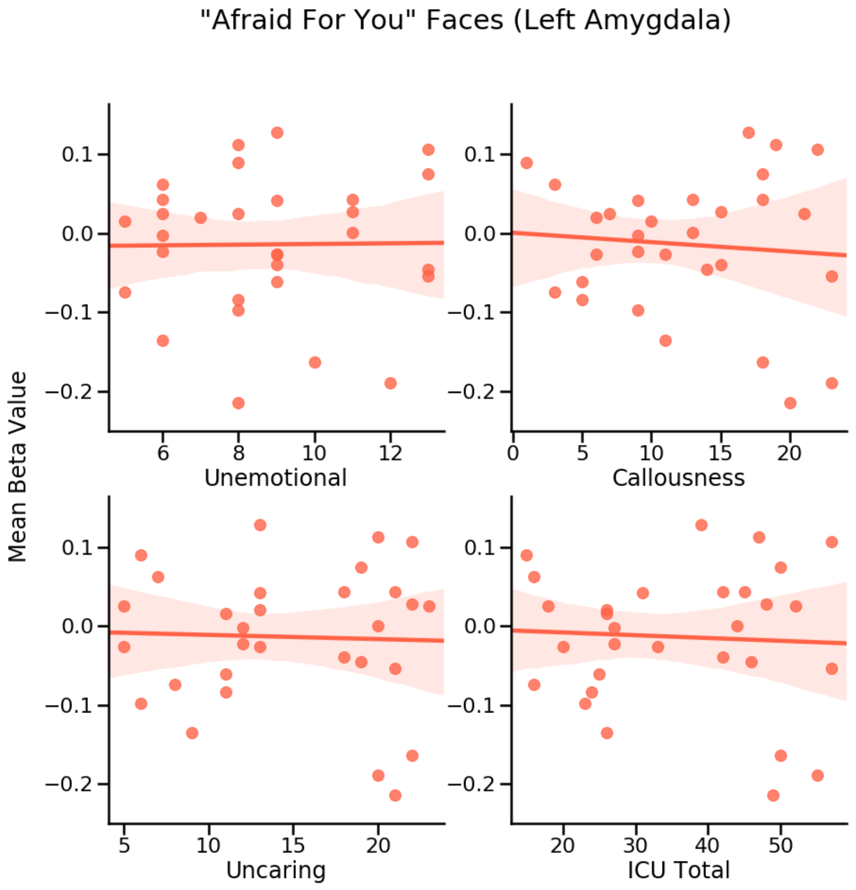
"Afraid For You" Faces (Left Amygdala)
Mean Beta Value
Unemotional
Callousness
Uncaring
ICU Total

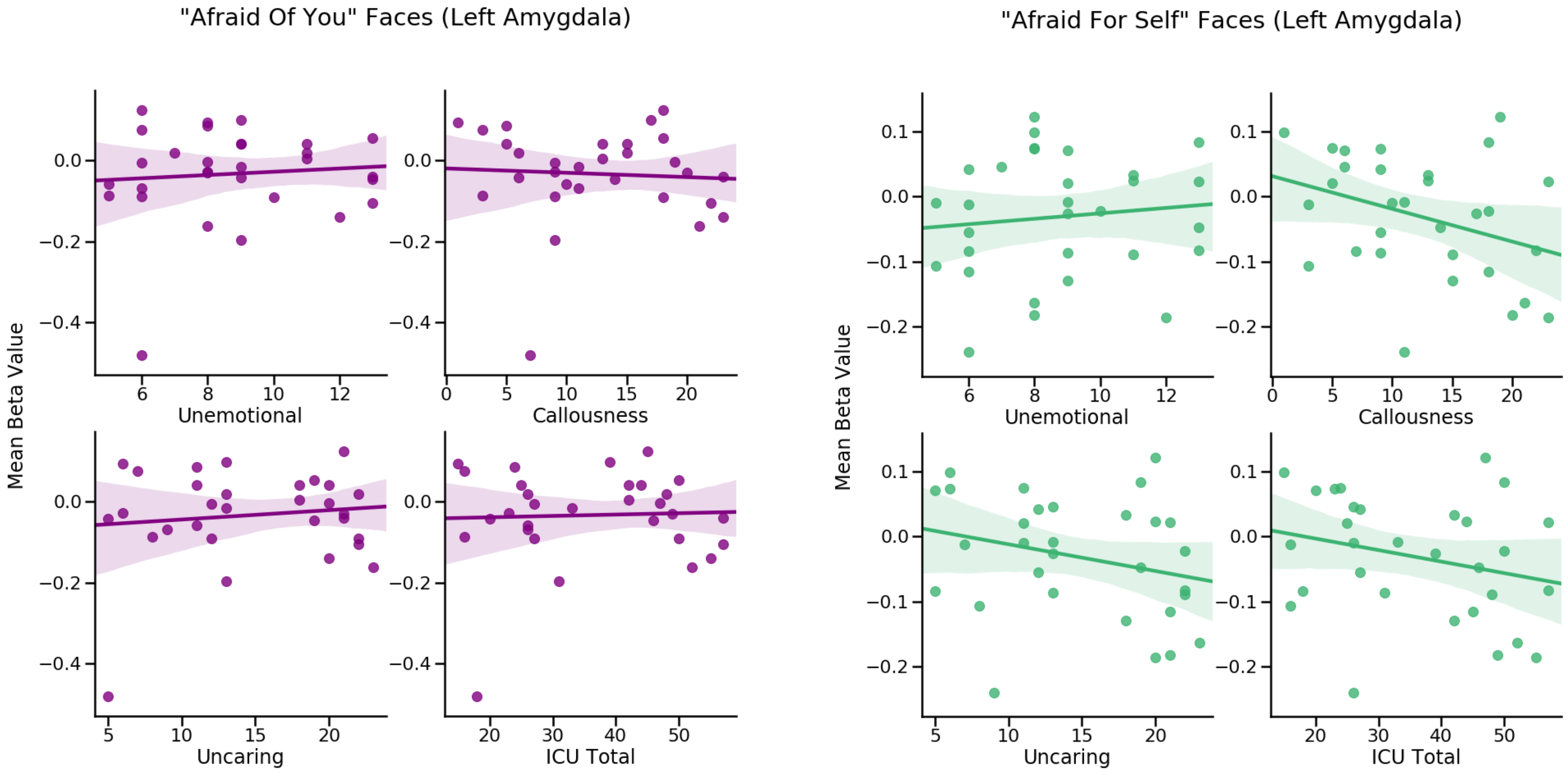
"Afraid Of You" Faces (Left Amygdala)
"Afraid For Self" Faces (Left Amygdala)
Mean Beta Value
Unemotional
Callousness
Uncaring
ICU Total

Supplementary Figure 2. Comparison of 30 subject x 30 subject inter-subject dissimilarity models across ICU subscales (callous, uncaring, unemotional) to inter-subject dissimilarity models based on ICU total scores.

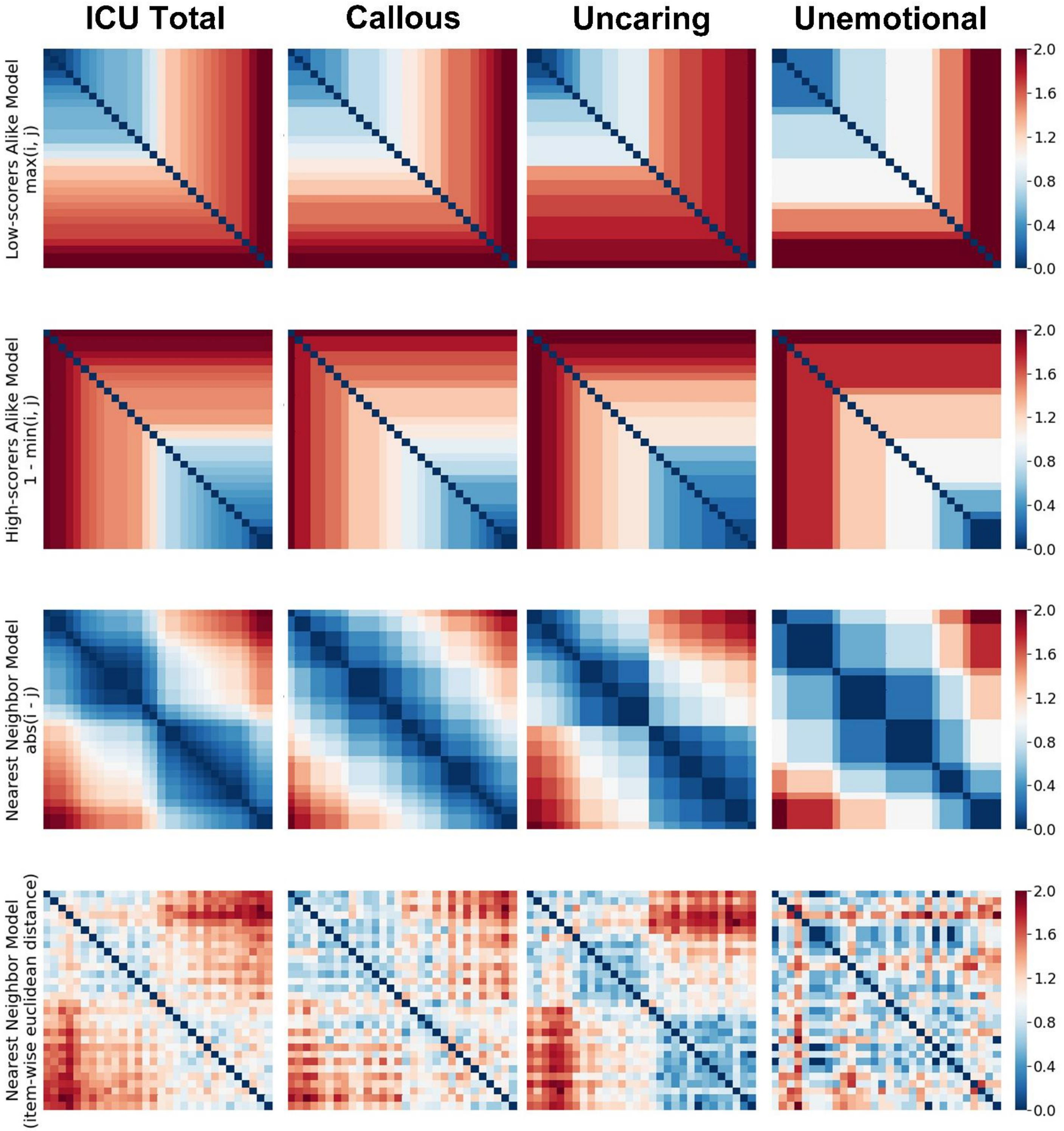

Note. Subjects are ordered in same ascending order based on their ICU total scores.
